# Genomic prostate score and treatment selection in favourable intermediate‐risk prostate cancer

**DOI:** 10.1002/bco2.494

**Published:** 2025-03-17

**Authors:** Eric Margolis, Benjamin H. Lowentritt, Christopher M. Pieczonka, John P. Bennett, Marina Pavlova, Joao Paulo Zambon, Jack Groskopf, Edward Uchio

**Affiliations:** ^1^ Hackensack Meridian School of Medicine Nutley NJ USA; ^2^ Chesapeake Urology Towson MD USA; ^3^ Associated Medical Professionals of NY Syracuse NY USA; ^4^ Exact Sciences Corporation Redwood City CA USA; ^5^ MDx Health Corporation Irvine CA USA; ^6^ University of California Orange CA USA

**Keywords:** active surveillance, biomarkers, Favourable intermediate‐risk, genomic testing, prostate cancer

## Abstract

**Objective:**

To assess the factors associated with the use of active surveillance (AS) in NCCN favourable intermediate‐risk (FIR) prostate cancer (PCa) patients who received the 17‐gene Genomic Prostate Score (GPS) assay.

**Material and Methods:**

Contemporary data were collected from academic and large community group practices across the United States. Eligible patients had localized PCa classified as FIR per NCCN guidelines and received a GPS report between May 2017 and April 2019. Higher GPS results (scale: 0–100) were associated with a higher risk of adverse outcomes. The proportion of patients selecting AS was calculated with 95% confidence intervals. Uni‐and multivariable logistic regression analyses were performed to determine the association between AS selection and relevant covariates.

**Results:**

There were 324 eligible patients (Gleason Score 3 + 4, 79%; PSA 10–20 ng/ml, 19%; clinical stage T2b‐T2c, 2%; median percent positive cores, 16.7%; median GPS result, 26). The distribution of GPS results was 0–19 (23%), 20–40 (60%), and 41–100 (16%). Overall, 31% (95% CI 26%, 36%) selected AS: 58% (46%, 69%) with GPS 0–19, 27% (21%, 33%) with GPS 20–40, and 6% (1%, 16%) with GPS 41–100. In univariable models, the Gleason score, percent positive cores, PSA, and GPS results were significantly associated with AS selection. In a multivariable model, the percent positive cores and the GPS result remained significantly associated with AS selection. AS persistence was 91% (82%, 95%) at 12 months.

**Conclusions:**

The GPS result and percent positive cores appear associated with AS use after controlling for relevant clinical variables in NCCN FIR prostate cancer patients.

## 1 INTRODUCTION

Prostate cancer (PCa) is the most common cancer in men after non‐melanoma skin cancer. In 2023, over 288 300 new cases and 34 700 deaths from PCa are estimated in the United States. Prostate cancer is also the second leading cause of cancer death in men and cancer treatment‐related years lived with disability worldwide, reflecting the confluence of its high incidence, long natural history, and treatment‐associated morbidity.^1^, ^2^

Accurate risk stratification of men with localized PCa is paramount for choosing the optimal treatment. The National Comprehensive Cancer Network (NCCN) uses clinical and pathological factors, including tumour stage, Gleason pattern, grade group, PSA and percent positive cores, to stratify patients into very low‐, low‐, favourable intermediate‐, unfavourable intermediate‐, high‐ and very high‐risk groups.^3^

The current treatment recommendations for most men with FIR disease include external beam radiation therapy (EBRT) or brachytherapy and radical prostatectomy with pelvic lymph node dissection. Active surveillance may be considered for those patients in the FIR group with a low percentage of Gleason pattern 4 cancer and low tumour volume. Notably, patients in the FIR stratification group vary considerably in their disease aggressiveness due to sampling error and biological potential. Distinguishing among these patients is critical for offering AS primarily to those patients with less aggressive diseases.^3^, ^4^

Studies performed on tumour‐based genomic assays have demonstrated they provide additional prognostic information independent of baseline clinical and pathological factors. Prostate cancer guidelines, including NCCN, AUA, ASCO and EAU, recommend using genomic assays when they have the potential ability to change management.^3^, ^5^, ^6^, ^7^ The GPS assay is a prospectively validated tool that aids treatment decision‐making by predicting the risk of adverse pathology [defined by high‐grade disease (primary Gleason pattern 4 or any pattern 5) and/or non‐organ confined disease (pathologic stage ≥ T3a)], biochemical recurrence, distant metastasis and prostate cancer death in patients with very low‐, low‐, intermediate‐ and high‐risk prostate cancer^8^, ^9^, ^10^, ^11^

This study focuses on patients with FIR PCa and assesses treatment decisions and outcomes in a cohort who received the GPS assay. We report the proportion and factors of selecting AS over definitive treatment, the results of a descriptive analysis on treatment selection, treatment‐related complications, and AS persistence. We hypothesized that the GPS result would be independently associated with AS in FIR patients.

## 2 MATERIAL AND METHODS

### 2.1 Patients and study design

We conducted a multicenter retrospective observational study at six community urology practices and one academic centre in the United States. Based on contemporary data obtained via commercial orders of the GPS assay, the first 60 patients (aged 18–89 years) at each study site with NCCN FIR prostate cancer and a GPS report dated between May 2017 and April 2019 were included. Patients were excluded from the analysis if they did not have the GPS result on the chart, had no record of a treatment decision, or had a previous GPS result. Data were collected by reviewing each study site's patient chart or electronic medical record. Data captured included demographic, clinicopathologic variables at PCa diagnosis, the GPS result and post‐GPS assay treatment decision. In addition, outcomes data up to the most recent encounter with the patient was captured, including PCa treatment‐related complications, persistence of AS, evidence of distant metastases, and prostate cancer‐related death. As this observational study aimed to record practice patterns at different study sites, no standardized, study‐wide AS protocol existed.

Additionally, no information was collected on additional biopsies, PSA results, or imaging during the post‐diagnosis surveillance period. The sites also reported whether the patients received a diagnosis of erectile dysfunction, urinary incontinence, or bowel incontinence at the time of PCa diagnosis and whether the patients had received a new diagnosis or had experienced a worsening of existing symptoms after treatment or the initiation of active surveillance. All patient data were anonymized before they were entered into the analysis dataset.

### 2.2 Endpoints

The primary endpoint was the proportion of patients who selected AS as initial PCa management and the determinants of this treatment decision. Secondary endpoints included the association between GPS results and treatment intensity and the association between treatment and treatment‐related complications. Exploratory endpoints included 12‐month AS persistence.

### 2.3 Statistical analyses

The statistical analysis plan was pre‐specified. Descriptive statistics were used to summarize clinicopathologic characteristics and patient outcomes, including prostate‐cancer‐related death, metastases, and treatment complications up to the patient's most recent encounter with the urologist. We determined the percentage of patients who selected AS over immediate definitive treatment and calculated 95% CIs using the Clopper‐Pearson method. Treatment intensity was categorized as AS, monotherapy, or multimodal therapy, and the percentage of patients receiving each was calculated with Clopper‐Pearson 95% CI and stratified by GPS result and relevant clinical factors (age, race/ethnicity, Gleason score, PSA, clinical T‐stage, percent positive cores). Based on prior research, we categorized the GPS result using cut points at 20 and 40.^9^, ^10^ For patients on AS, time from GPS report to delayed treatment was calculated, and the percentage of patients remaining on AS at 12 months was determined using the Kaplan–Meier method. Patients who were persistent on AS were censored at the date of their most recent encounter with the study site. A post hoc logistic regression analysis was performed on the association between AS selection and GPS result, along with relevant clinical covariates. Univariable and multivariable models were evaluated, and odds ratios (ORs) with 95% confidence intervals were reported. Analyses were performed using SAS software version 9.4 of the SAS System for Windows and R version 4.0.1.^12^ Graphics were created using the ggplot2 package.^13^

## 3 RESULTS

### 3.1 Study cohort

**FIGURE 1:**
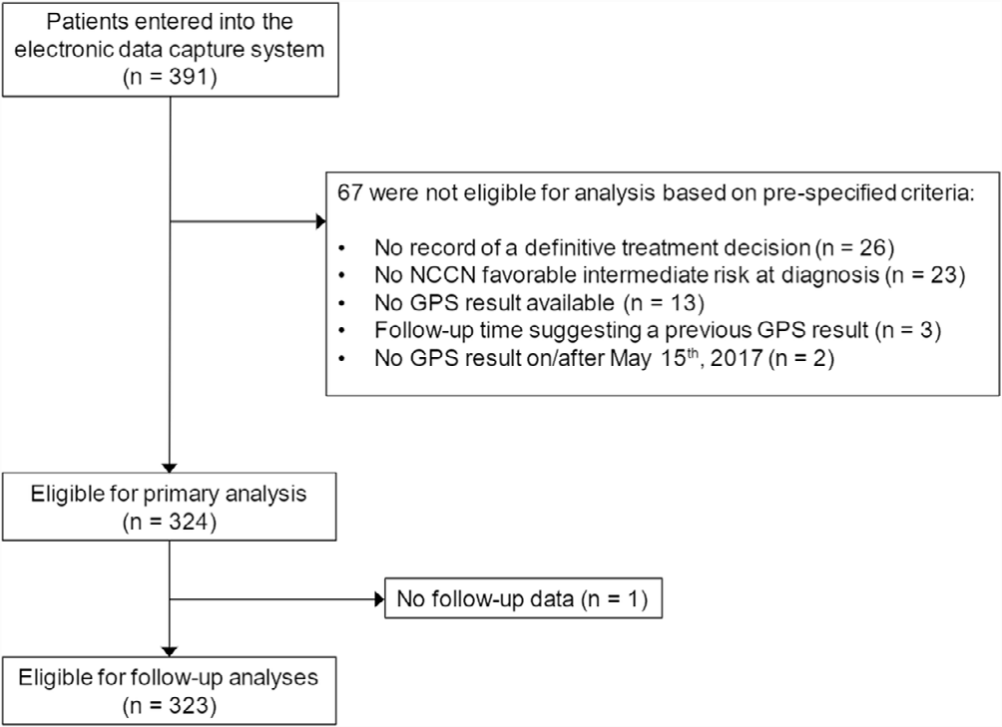
Patient disposition.

Data were collected for 391 NCCN FIR PCa patients at the seven study sites. Based on pre‐specified criteria, 324 (83%) were eligible for primary analysis (Figure 1). One patient had no post‐treatment data, and thus, the follow‐up analyses included 323 patients. Patient demographics and clinicopathologic tumour characteristics at PCa diagnosis are summarized in Table 1. The median patient age was 67 (interquartile range, 61–71) years, the majority (62%) was White, 15% of patients were Hispanic/Latino, and 12% were Black/African American. According to NCCN guidelines, FIR patients can have one intermediate‐risk factor; in this cohort, the vast majority (79%) had biopsy Gleason score of 3 + 4, whereas 19% had PSA 10–20 ng/ml, and just 2% had clinical stage T2b (0 had clinical stage T2c). A median of 12 biopsy cores was collected (range 3 to 48); 54% had ≤ 16.7% positive cores (or 1 to 2 positive cores out of 12 collected). The median GPS result was 26 (range 0–100). 23% of patients had GPS results between 0 and 19, 60% between 20 and 40, and 16% between 41 and 100.

**TABLE 1:** Patient and tumour characteristics at prostate cancer diagnosis. P‐values are from chi‐square and Fisher's exact tests.

Variable	All (N = 324)	Active surveillance (N = 99)	Immediate treatment (N = 225)	p‐value
**Patient age (y)**				0.11
Median (interquartile range)	67 (61 to 71)	66 (60 to 72)	67 (62 to 71)	
Range	46 to 89	50 to 83	46 to 89	
< 55	20 (6%)	3 (3%)	17 (8%)	
55–64	102 (31%)	37 (37%)	65 (29%)	
65–74	160 (49%)	43 (43%)	117 (52%)	
≥ 75	42 (13%)	16 (16%)	26 (12%)	
**Race/ethnicity, n (%)**				0.17
Hispanic or Latino	48 (15%)	20 (20%)	28 (12%)	
Non‐Hispanic/Latino				
White	200 (62%)	59 (60%)	141 (63%)	
Black or African American	40 (12%)	12 (12%)	28 (12%)	
Asian	3 (1%)	1 (1%)	2 (1%)	
Native Hawaiian or other Pacific Islander	1 (<1%)	1 (1%)	0	
Unknown	32 (10%)	6 (6%)	26 (12%)	
**Gleason score, n (%)**				<0.001
3 + 3	69 (21%)	33 (33%)	36 (16%)	
3 + 4	255 (79%)	66 (67%)	189 (84%)	
**PSA**				0.002
Median (interquartile range), ng/mL	6.2 (4.6 to 8.6)	7.0 (4.9 to 10.2)	6.0 (4.5 to 8.0)	
Range, ng/mL	0.1 to 20.0	0.1 to 20.0	0.4 to 20.0	
< 10 ng/ml	262 (81%)	70 (71%)	192 (85%)	
≥ 10 ng/ml	62 (19%)	29 (29%)	33 (15%)	
**PSA density** ^**a**^				0.95
Median (interquartile range), ng/cm^3^	0.15 (0.10 to 0.21)	0.14 (0.09 to 0.21)	0.15 (0.10 to 0.21)	
Range, ng/cm^3^	0.01 to 0.80	0.01 to 0.48	0.02 to 0.80	
< 0.15 ng/cm^3^	159 (51%)	49 (52%)	110 (51%)	
≥ 0.15 ng/cm^3^	151 (49%)	46 (48%)	105 (49%)	
**Clinical stage, n (%)**				0.053
cT1c	290 (90%)	91 (92%)	199 (88%)	
cT2a	27 (8%)	4 (4%)	23 (10%)	
cT2b	7 (2%)	4 (4%)	3 (1%)	
cT2c	0			
**Percent positive cores**				0.004
Median (interquartile range)	17% (8% to 27%)	17% (8% to 25%)	20% (14% to 33%)	
Range	2% to 47%	2% to 42%	2% to 47%	
≤ 16.7%	174 (54%)	65 (66%)	109 (48%)	
> 16.7%	150 (46%)	34 (34%)	116 (52%)	
**GPS result**				<0.001
Median (interquartile range)	26 (20 to 35.5)	21 (15 to 30)	29 (22 to 39)	
Range	0 to 100	0 to 52	1 to 100	
0–19	76 (23%)	44 (44%)	32 (14%)	
20–40	195 (60%)	52 (53%)	143 (73%)	
41–100	53 (16%)	3 (3%)	50 (22%)	

^a^ PSA density was missing in 14 patients.

### 3.2 Therapy selection

**FIGURE 2:**
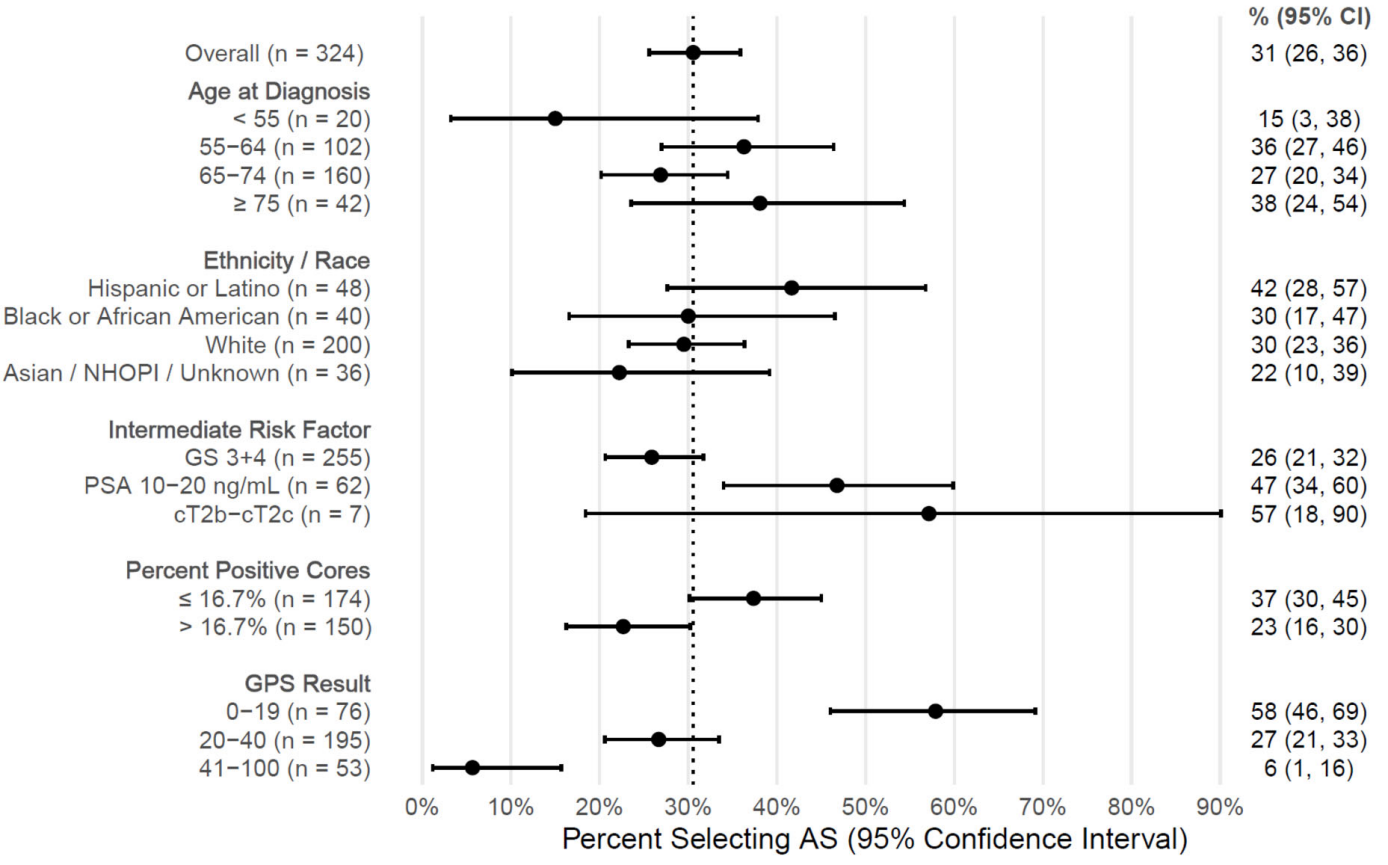
Percent of patients selecting AS (with 95% CI), overall and by relevant clinical factors and GPS result. The dashed line is the percentage of patients selecting AS (31%).

Overall, 99 out of 324 patients (31%; 95% CI 26%, 36%) initially selected AS. The percentages of patients selecting AS are presented in Figure 2. Slight differences in AS selection percentages were noted between demographic subgroups (age, ethnicity/race), but confidence intervals overlap. AS selection was lower for patients with biopsy Gleason score 3 + 4 than those with PSA 10–20 ng/ml (26% vs. 47%, Figure 2). There was an insufficient number of patients with clinical stage T2b‐2c to determine whether their AS selection percentages differed from that of patients with the other two intermediate risk factors.

Patients with a low percentage of positive cores (≤16.7%) selected AS more frequently than those with a higher percentage of positive cores (37% vs. 23%). Concerning the GPS assay, AS selection declined with higher GPS values: the percentage of AS selection was 58% (95% CI 46% to 69%) for patients with GPS results 0–19, 27% (95% CI 21% to 33%) for GPS results 20–40 and 6% (95% CI 1% to 16%) for GPS results 41–100 (p < 0.001). The association between the GPS values and AS is demonstrated in Figure 2. In a subset analysis on the Gleason score 3 + 4 patients (n = 255), AS selection was reported in 50% (95% CI 35% to 65%) of patients with GPS results 0–19, 26% (19% to 33%) of patients with GPS result 20–40 and 6% (1% to 16%) of patients with GPS result 41–100.

**FIGURE 3:**
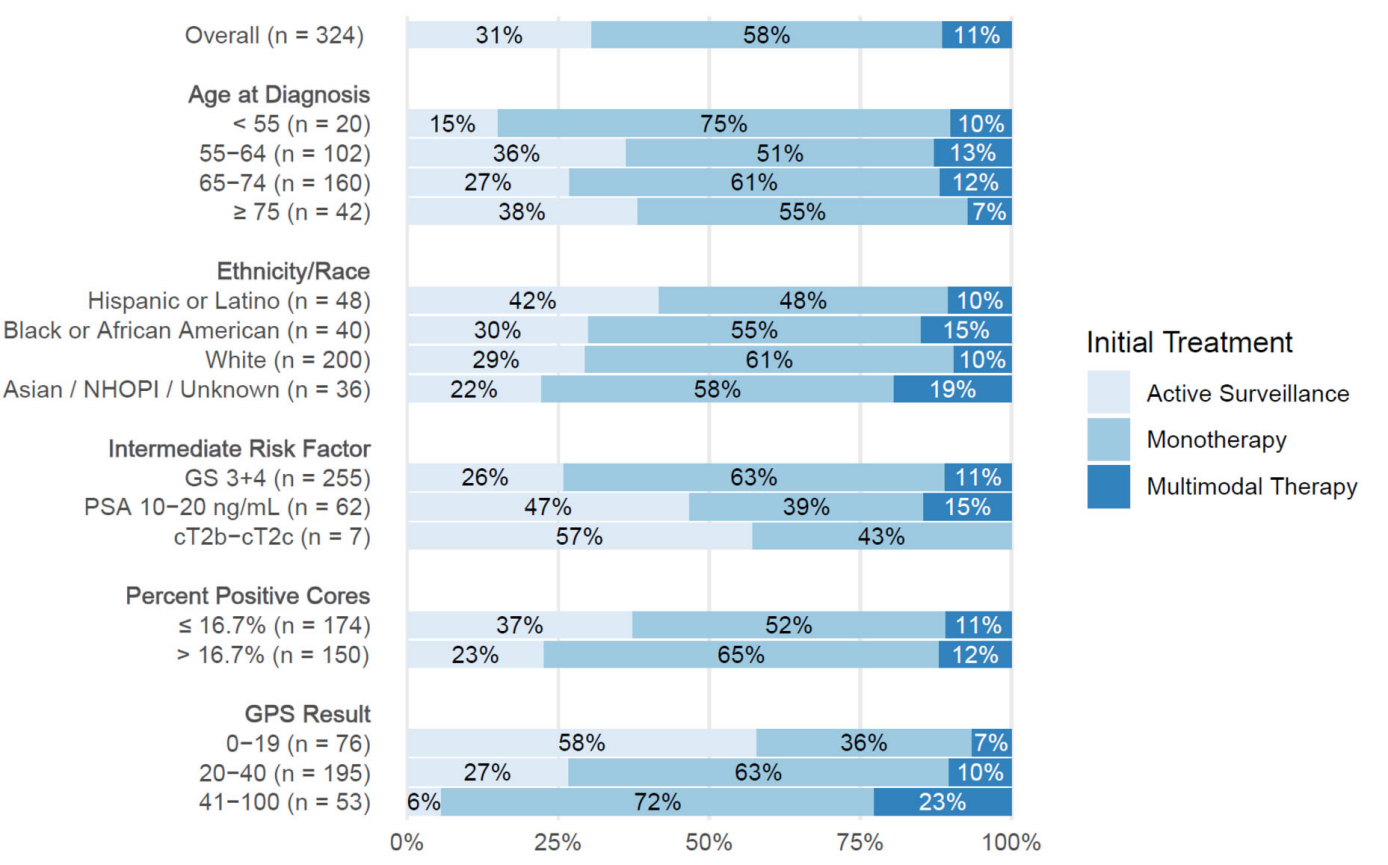
Treatment intensity, overall, and by relevant clinical variables and GPS result. Monotherapy: RP, EBRT, ADT, focal therapy, or other multimodal therapy: EBRT plus ADT, RP plus EBRT, brachytherapy plus ADT.

A detailed summary of the selected treatments is presented in Table 2. Overall, 188 patients (58%) were treated with monotherapy and 37 (11%) with multimodal therapy. The most common monotherapy was EBRT (88/188, 47%), followed by RP (70, 37%) and the most common multimodal therapy was EBRT plus ADT (33/37, 89%). Analysis of initial treatment intensity (AS, monotherapy, multimodal therapy) by GPS result showed the percentage of patients receiving monotherapy was higher in GPS result ranges 20–40 (63%) and 41–100 (72%) versus 0–19 (36%), and the proportion of patients receiving multimodal therapy was highest in the GPS result range 41–100 (GPS result 41–100, 23%; 20–40, 10%; and 0–19, 7%) (Figure 3).

**TABLE 2:** Initial therapy by treatment intensity, N = 324.

Active surveillance (N = 99, 31%)	
Monotherapy (N = 188, 58%)	
**Treatment:**	**N (%)**
EBRT	88 (47%)
RP	70 (37%)
ADT	10 (5%)
Focal therapy	10 (5%)
Brachytherapy	9 (5%)
Other	1 (1%)
**Multimodal therapy (N = 37, 11%)**	
**Treatment:**	**N (%)**
EBRT + ADT	33 (89%)
RP + EBRT	3 (8%)
Brachytherapy + ADT	1 (3%)

EBRT = External beam radiation therapy, ADT = Androgen deprivation therapy, RP = Radical Prostatectomy.

The ORs for selecting AS for initial PCa management are presented in Table 3. Gleason score, percent positive cores, PSA, and GPS results were statistically significant covariates in univariable models. In the multivariable model, the GPS result remained significantly associated with AS selection (OR for GPS result per 20 units: 0.27 [95% CI 0.14, 0.39], p < 0.001) as did percent positive cores (OR per 1 standard deviation: 0.27 [0.16, 0.45], p < 0.001).

**TABLE 3:** Univariable and multivariable logistic regression models evaluate the association between AS selection (vs. immediate treatment) and relevant covariates (N = 324).

Covariate	No. AS/total	Univariable		Multivariable	
		OR (95% CI)	p	OR (95% CI)	p
Age at diagnosis (y)	99/324	1.01 (0.79, 1.28)	0.960	1.08 (0.83, 1.42)	0.561
Race/ethnicity			0.261		0.346
White	59/200	Reference		Reference	
African American	12/40	1.02 (0.47, 2.11)		0.87 (0.37, 1.97)	
Hispanic or Latino	20/48	1.71 (0.88, 3.26)		1.83 (0.89, 3.74)	
Asian/NHOPI/Unk	8/36	0.68 (0.28, 1.52)		0.91 (0.35, 2.22)	
Log PSA (ng/mL)	99/324	1.17 (0.92, 1.53)	0.221	0.87 (0.62, 1.20)	0.388
Clinical stage			0.078		0.469
T1c	91/290	Reference		Reference	
T2a	4/27	0.38 (0.11,1.02)		0.49 (0.13, 1.42)	
T2b	4/7	2.92 (0.63,15.05)		1.05 (0.15, 7.64)	
Gleason score			<0.001		0.163
3 + 3	33/69	Reference		Reference	
3 + 4	66/255	0.38 (0.22, 0.66)		0.56 (0.25, 1.27)	
Percent positive cores	99/324	0.62 (0.48,0.80)	<0.001	0.71 (0.53,0.94)	0.019
GPS result per 20 u	99/324	0.24 (0.14,0.39)	<0.001	0.27 (0.16,0.45)	<0.001

AS = Active surveillance, OR = odds ratio, CI=Confidence interval, NHOPI = Native Hawaii and Other Pacific Islander, Unk = Unknown, PSA = prostate‐specific antigen. The outcome in all models is AS selection. ORs < 1 are associated with less AS selection, and ORs > 1 are associated with more AS selection. Variables for age, log PSA, and percent positive cores are standardized. Consistent with prior analyses, the GPS result is reported per 20 units.

### 3.3 Post‐treatment outcomes

Among the 323 patients with follow‐up data, the median time (interquartile range) from the GPS report date to the most recent encounter with the patient was 18 (14, 24) months. A patient with a GPS result of 55 was diagnosed with bone metastasis two months after the GPS report; 283 patients (88%) reported no prostate cancer‐related metastasis, and the metastasis status was unknown for the remaining 39 patients (12%). Two deaths were recorded (patients aged 72 and 74 years), and neither was determined to be related to prostate cancer.

A higher proportion of patients receiving therapy had prostate cancer‐related complications (erectile dysfunction, urinary incontinence and bowel incontinence) than those who went on AS. Patients who underwent radical prostatectomy monotherapy had the most significant increases in pre‐ to post‐treatment diagnosis of erectile dysfunction (23% pre‐treatment to 64% post‐treatment) and urinary incontinence (4% pre‐treatment to 40% post‐treatment).

AS persistence was 91% (95% CI 82% to 95%) at 12 months. The median (interquartile range) follow‐up time among the patients who remained on AS was 16.5 (12 to 22) months. In total, 18 patients discontinued AS during the follow‐up period; 8 had EBRT, 7 had radical prostatectomy, 1 had multimodal therapy, 1 had focal therapy, and 1 had unknown therapy. Also, of these 18 patients, 11 discontinued AS due to documented disease progression; the remaining seven discontinued AS due to patient preference or were discontinued for unknown reasons.

## 4 DISCUSSION

Even as AS is becoming more widely accepted as an option for patients with FIR prostate cancer, there is a lack of consensus on a standard of care for these patients and perhaps a role for molecular biomarkers to aid in identifying patients who are candidates for this management strategy.^12^, ^13^ The percentages of AS use by clinicopathologic factors and the GPS results (Figure 2) suggest biopsy Gleason score 3 + 4, positive cores >16.7% (equivalent to > 2 positive cores in a standard 12‐core biopsy), and GPS results 41–100 are associated with lower AS use. Patients with a higher PSA (10–20 ng/ml) had relatively high AS use (47%), which can be attributed to the criteria for determining which patients have FIR disease: they can have only a single intermediate risk factor, so all patients with PSA values between 10 and 20 ng/ml would have also had Gleason score 3 + 3 biopsies. Gleason's Score appears to be more strongly associated with AS use than PSA. The demographic factor examined (age, ethnicity/race) did not appear to be associated with AS use.

The univariable logistic regression models in Table 3 show the same factors associated with AS use as in Figure 2. PSA density is not included in Figure 2 but was not significantly associated with AS use in an univariable model. In the multivariable model, only the GPS result and percent positive cores were found to be associated with AS use. Diagnostic PSA and biopsy Gleason score, which appeared to be strongly associated with AS use in the univariable model, were not statistically significant after controlling for the other covariates. Note that NCCN criteria for FIR limit positive cores to <50%, which, for a standard 12‐core systematic biopsy, is between 1 and 5 positive cores.^3^ Even with this limited range, there does appear to be a difference in AS use between patients with 1–2 positive cores (2 positive cores out of 12 total cores is 16.7%) vs. 3–5 positive cores (>16.7%). Percent positive cores have previously been shown to be associated with pathological upgrading in clinically low‐risk disease, which could explain its presence as an independent factor in AS selection in this cohort.^14^ After controlling for relevant covariates, the GPS result remained independently associated with AS selection. Patients with lower GPS results tended to select AS, whereas patients with higher GPS results tended to receive definitive treatment. The vast majority of AS patients persisted on AS for at least one year. Regarding treatment intensity, the GPS result in the range 41–100 had the highest percentage of patients receiving multimodal therapy (23%) and the lowest percentage on AS (6%) out of all other factors, including percent positive cores.

Also, as expected, prostate cancer‐related complications were more common among patients receiving treatment than those on AS. Notably, only one prostate cancer‐related metastasis and two deaths (unrelated to prostate cancer) were reported during the study period.

Before AS was added to guidelines as consideration for FIR prostate cancer, its use was increasing annually in this risk group in the US, from 7.2% in 2010 to 14.9% in 2015.^15^ The higher percentage of AS use observed in this cohort (31%) can be partially attributed to the later time period (2017–2019), which occurred after the change to guidelines but could also be due to the GPS assay being ordered by physicians for patients who they considered for AS. Therefore, the AS percentage observed in this cohort is not generalizable to all patients with FIR prostate cancer but to the subset who received a genomic assay before making an initial treatment decision. Prior clinical utility studies also demonstrated that the GPS result increased physicians' confidence and decreased patients' decisional conflict.^16^, ^17^, ^18^, ^19^, ^20^ The current study did not evaluate these parameters, and additional studies focusing on these aspects in NCCN FIR patients are warranted.

The strengths of our study include its representation of real‐life clinical practice at academic and community‐based practices and several geographically distinct locations in the United States. Thus, our study represents clinical practice in a diverse patient population with a broad spectrum of treatments. Our study is limited by its retrospective observational design, limited follow‐up time, and limited data on surveillance biopsy and multiparametric MRI scans in patient care. While this study focused on the association of a genomic assay with therapy selection, it would also be helpful to study long‐term outcomes for these patients.

## 5 CONCLUSIONS

GPS results and percent positive cores were independently associated with AS use in a contemporary cohort of FIR prostate cancer patients who received a GPS assay. Patients with a lower GPS result and a lower percentage of positive cores were more likely to select AS as initial disease management.

## AUTHOR CONTRIBUTIONS

**Eric Margolis:** Study design, data acquisition, data analysis, article writing, and review. **Benjamin Lowentritt:** Data acquisition, data analysis, article writing, and review. **Cristopher Pieczonka:** Data acquisition, data analysis, article writing, and review. **John P. Bennett:** Statistical analysis. **Marina Pavlova:** Statistical analysis. **Joao Paulo Zambon:** Data analysis, article writing, and review. **Jack Groskopf:** Article writing and review. **Edward Uchio:** Study design, data acquisition, data analysis, article writing, and review.

## CONFLICT OF INTEREST STATEMENT

Eric Margolis.

No conflict of interest.

Benjamin H. Lowentritt.

No conflict of interest.

Cristopher M Pieczonka.

Consultant of Astellas, Astra Zeneca, Bayer, Daiichi‐Sankyo, Dandreon, Janssen, Myovant, Merck, Pfizer, and Sun.

Research Funding: Invitae, Genome FX, and Exact Sciences.

John P. Bennett.

Full‐time employment at Exact Sciences Corporation.

Marina Pavlova.

Full‐time employment at Exact Sciences Corporation.

Joao Paulo Zambon.

Full‐time employment at mdx Health.

Jack Groskopf.

Full‐time employment at mdx Health.

Edward Uchio.

No conflict of interest.
